# Does different vertical position of maxillary central incisors in women with different facial vertical height affect smile esthetics perception?

**DOI:** 10.1186/s40510-023-00479-y

**Published:** 2023-08-07

**Authors:** Ezgi Atik, Hilal Turkoglu

**Affiliations:** Department of Orthodontics, Faculty of Dentistry, Hacettepe University, 06100 Sihhiye, Ankara, Turkey

**Keywords:** Esthetics, Facial height, Maxillary incisor, Smile, Vertical position

## Abstract

**Background:**

The aim of this study was to assess the esthetic perceptions of orthodontists, prosthodontists and laypersons with regard to different vertical positions of the maxillary central incisors related to lateral incisors for different facial vertical height cases.

**Subject and methods:**

Frontal full-face photographs showing social smiles of three adult women aged between 18 and 25 years were used. Vertical position of the maxillary central incisor was changed (intruded or extruded) with 0.5 mm increments according to the reference gingival line resulting five images for each woman in a full-face view yielding a total of 15 images. A visual analog scale was placed below each smile to allow the raters to evaluate the attractiveness of each smile independently. One-way analysis of variance was used to determine whether there was a difference between more than two independent groups in terms of quantitative variables. Comparisons of more than two dependent groups were examined with repeated measures one-way ANOVA. The significance level was taken as 0.05 for all analyses.

**Results:**

For increased facial vertical height, the highest scores for orthodontists were given to the 0.5 mm extruded (64.18 ± 26.36), for prosthodontists to the control (57.28 ± 19.80), and for layperson to the 1 mm extruded (61.27 ± 25.98) central incisor position. For decreased facial vertical height, the highest scores were obtained at the 0.5 mm intrusion with an increasing pattern from orthodontists to laypersons (63.95 ± 22.08 for orthodontists, 79.87 ± 21.43 for prosthodontists, and 79.88 ± 19.17 for laypersons). All three rater groups gave the highest scores to the 0 mm (control) smile design for normal facial vertical height. When these scores were compared among the groups, laypersons gave significantly higher scores compared to orthodontists (*p* < 0.001) and prosthodontists (*p* = 0.005).

**Conclusions:**

The facial vertical height significantly affected the perception of smile esthetics. Keeping the distance between the central and lateral incisors longer than 1 mm in individuals with increased facial height may be important in terms of increasing patient satisfaction in terms of clinical aesthetics. On the contrary, keeping the distance between the central and lateral incisors shorter than 1 mm may create a more esthetically acceptable result in individuals with short facial height.

## Background

The search for improved dentofacial esthetics is an important issue frequently encountered in modern societies. Therefore, patients who want a beautiful face and smile are looking for treatment methods that will improve dentofacial esthetics and provide a positive change to their smiles. The vertical position of the upper central incisors, which takes into account both the gingival margins and the incisal edges, is one of the important aspects of dentofacial esthetics [1]. Several studies have suggested that the maxillary central incisors and canines must be placed almost at the same level and that the incisal edge of the lateral incisors should be leveled 0.5–1.5 mm more gingivally [2–4].

Since small vertical modifications on maxillary central incisors would modify the perception of smile esthetics, it is important to be sure about the best vertical relationship between the lateral and central incisor edges for each patient to assist the clinician in optimizing smile esthetics during bracket positioning as well as the finishing and detailing phases [5]. Most contemporary protocols for bracket placement in orthodontics prescribe a difference of 0.5 mm [6, 7]. Yet many authors have suggested that an occlusal height difference greater than 0.5 mm between the maxillary central and the lateral incisor may improve the esthetics [1, 8, 9].

While planning an orthodontic treatment, the perception of dentofacial esthetics by laypersons and professionals, such as orthodontist or general dentists, should be considered [10]. Since esthetic treatment procedures frequently involve the anterior esthetic zone and require a multidisciplinary team approach, the different specialists involved should share their knowledge regarding smile esthetics among one another, which would facilitate communication and would improve the treatment outcome [4, 11]. There are several studies that have compared the opinions of orthodontists, general dentists, and lay people regarding smile esthetics [1, 4, 5, 12–18]. However, to the best of our knowledge, no study has compared the opinions of orthodontists, prosthodontists, and laypersons regarding the vertical positions of anterior teeth and smile esthetics.

Facial type (i.e., short face, long face, or normal face) is one of the important fundamental factors playing role in determining smile attractiveness [19]. Most of the studies evaluating the impact of vertical positions of incisors on smile attractiveness used only close-up images of smiles [1, 4, 20]. However, we believe in that studies evaluating smile attractiveness should also consider the facial type of the patient, since the vertical position of incisors in different facial types may appear different from an esthetical point of view.

The objective of this study was to assess the esthetic perceptions of orthodontists, prosthodontists, and laypersons regarding the different vertical positions of maxillary central incisors related to lateral incisors in different facial vertical height cases. This study was designed to fill a gap in the literature about the relationship between vertical facial dimensions and smile perceptions related to the vertical position of maxillary central incisors. The null hypothesis of the study was that the different vertical position of maxillary central incisors affects the perception of orthodontists, prosthodontists, and laypersons in the same way regardless of vertical facial dimension.

## Materials and methods

Ethical approval for this study was obtained from the Institutional Review Board of Hacettepe University with the number of GO 22/462. G*Power software (version 3.1.9.213; Heinrich Heine Universitat Dusseldorf Institute Experimentelle Psychologie, Dusseldorf, Germany) was used to calculate the study’s sample size. Considering an alpha error of 0.01, 80% power, and a 0.25 effect size, 60 raters per group would be enough for this research to be consistent with other studies that used similar methods [1, 21, 22]. The inclusion criteria for the raters were as follows: (1) between 25 and 65 years old; (2) both man and woman; (3) orthodontists should have at least two years of experience; (4) prosthodontists should have at least two years of experience; and (5) laypersons should have a university degree but have no training in dentistry before; they should not have received any orthodontic treatment in the previous five years and should not have any close contact with dentists.

Frontal full-face photographs showing the social smiles of three adult women (white Caucasian) aged between 18 and 25 years were used. The subjects were informed about the study, and signed informed consents were obtained from the subjects who were willing to participate. To evaluate the perception of smile esthetics, we used a full-face view instead of a close-up one. In this way, it could be possible to reveal whether facial height change significantly influences the vertical position of maxillary centrals in terms of aesthetic perception. All the features that could identify the people in the photographs were destroyed, and a black line was drawn on a part of the eyes to prevent their recognition. The participants included in this study were patients who had completed orthodontic treatments before in the orthodontic clinical department of Hacettepe University. Each case represented different facial vertical dimensions. Vertical facial development was assessed via lateral cephalometric radiographs. Sagittal skeletal malocclusion was the same (skeletal Class I malocclusion 2 < ANB° < 4), whereas the vertical measurements were different, according to work of Fields et al. [23]. (The first case represented a normal vertical facial dimension: 26 < GoGnSN° < 38, 64 < ANS-Me(mm) < 72; the second case represented a decreased vertical facial dimension: GoGnSN° < 26, ANS-Me(mm) < 64; and the third case represented an increased vertical facial dimension: GoGnSN° > 38, ANS-Me(mm) > 72 mm.). The inclusion criteria for the patients were as follows: (1) they had healthy maxillary anterior dentition; (2) they had undergone no restorative procedures; (3) they had no crowding or spacing in the maxillary anterior region; (4) they had no facial asymmetry recognized by clinical examination; and (5) they had no history of orthognathic and prosthetic treatment. The full-face facial photographs of three women who were informed about the study and willing to participate were used. During the photograph taking, the patients were asked to sit on a chair and put their head in a natural head position. The camera was mounted on a stand about 90 cm from the patient so that it was equal to the height of the patient’s face. The camera looked into the patient’s eyes. At the same time, they were asked to look at a mirror placed opposite to them. After obtaining a reproducible smile [24], images were taken with a social smile position, which exposed the distal aspect of the canine teeth.

**Fig. 1:**
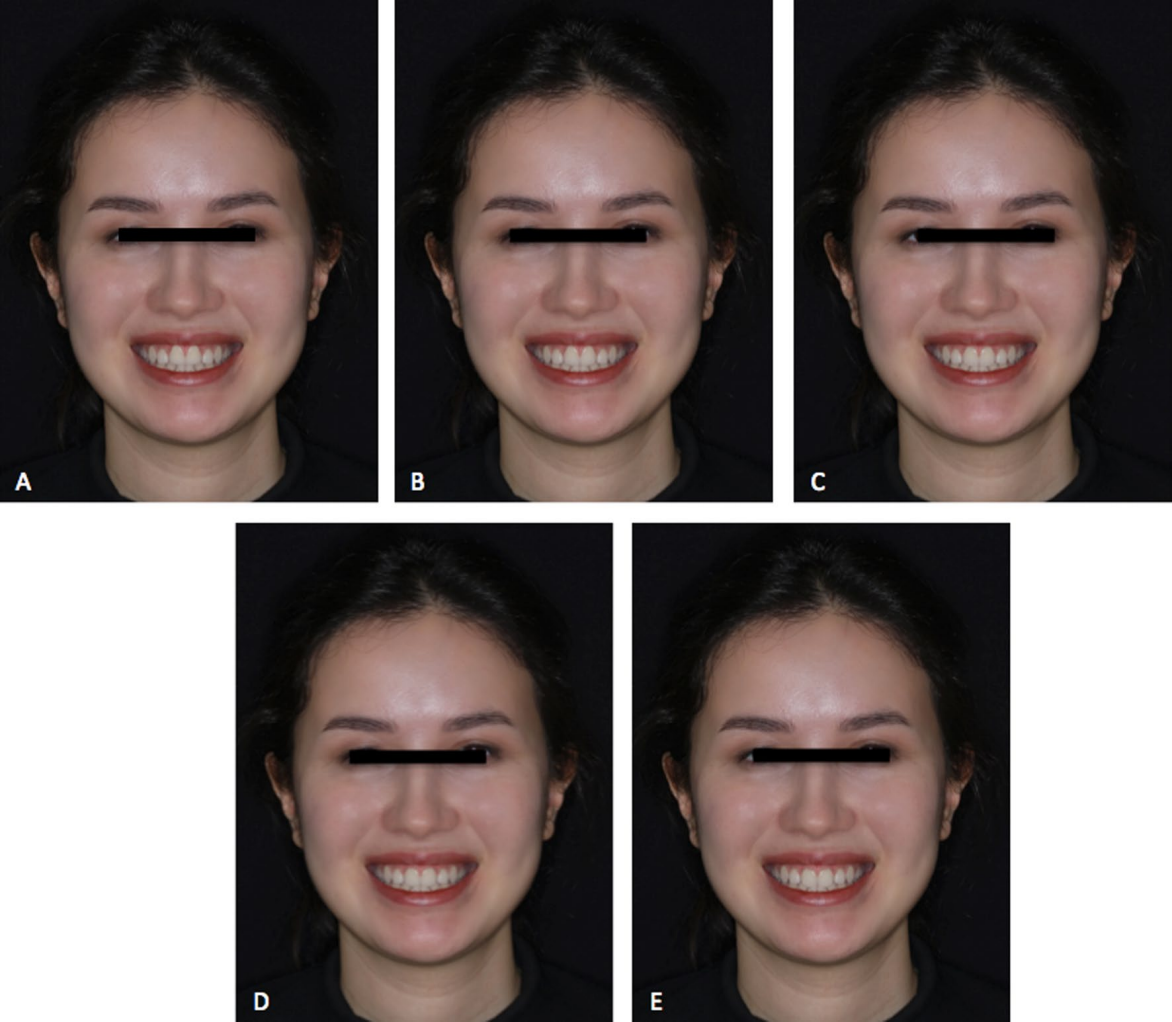
Full-face smile view in 0.5 mm altered vertical positions increments of a white woman showing normal vertical facial dimension. **A **− 1 mm; **B** − 0.5 mm; **C** 0 mm; **D** + 0.5 mm; **E** + 1 mm

**Fig. 2:**
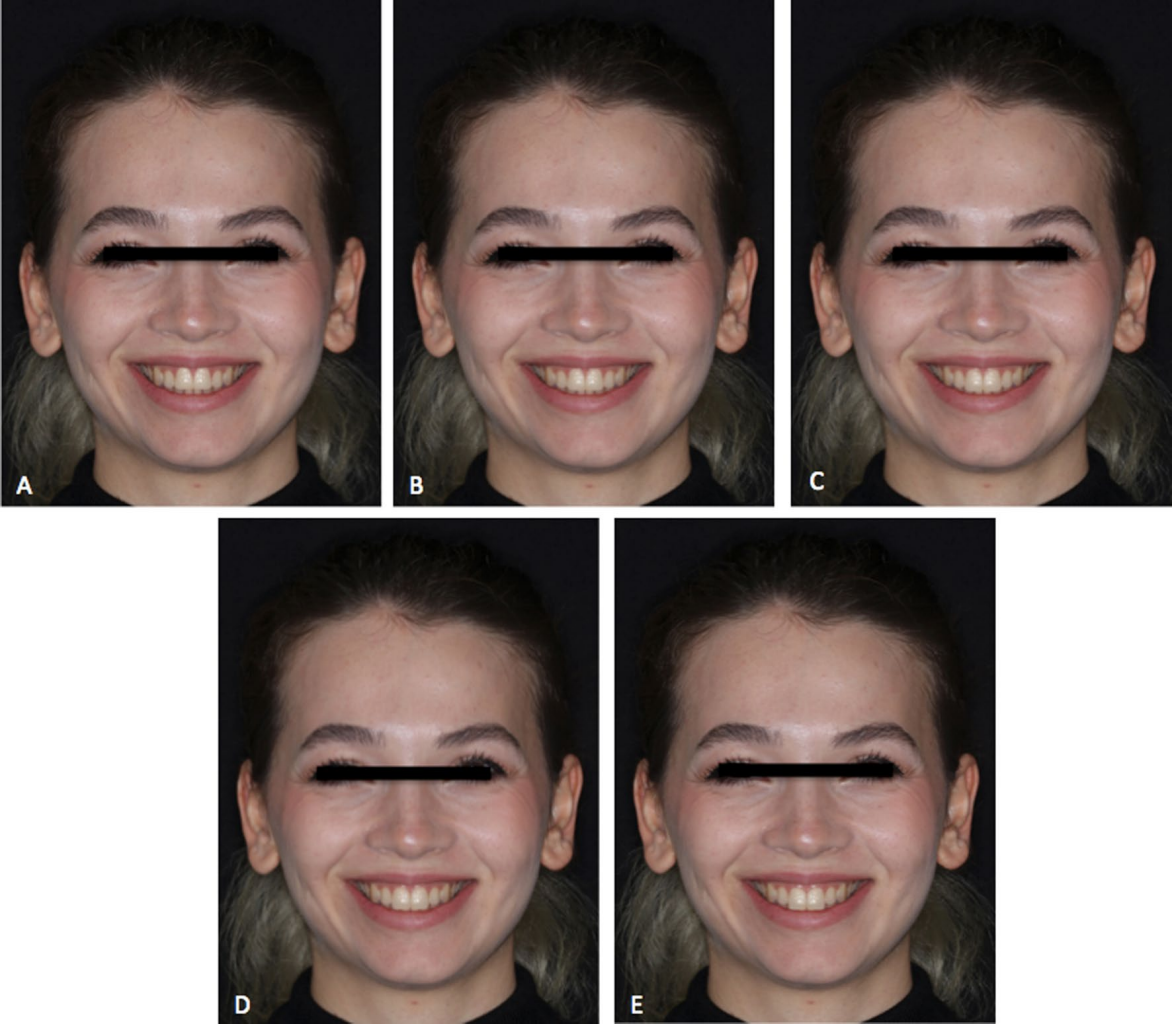
Full-face smile view in 0.5 mm altered vertical positions increments of a white woman showing decreased vertical facial dimension. **A** − 1 mm; **B** − 0.5 mm; **C** 0 mm; **D** + 0.5 mm; **E** + 1 mm

**Fig. 3:**
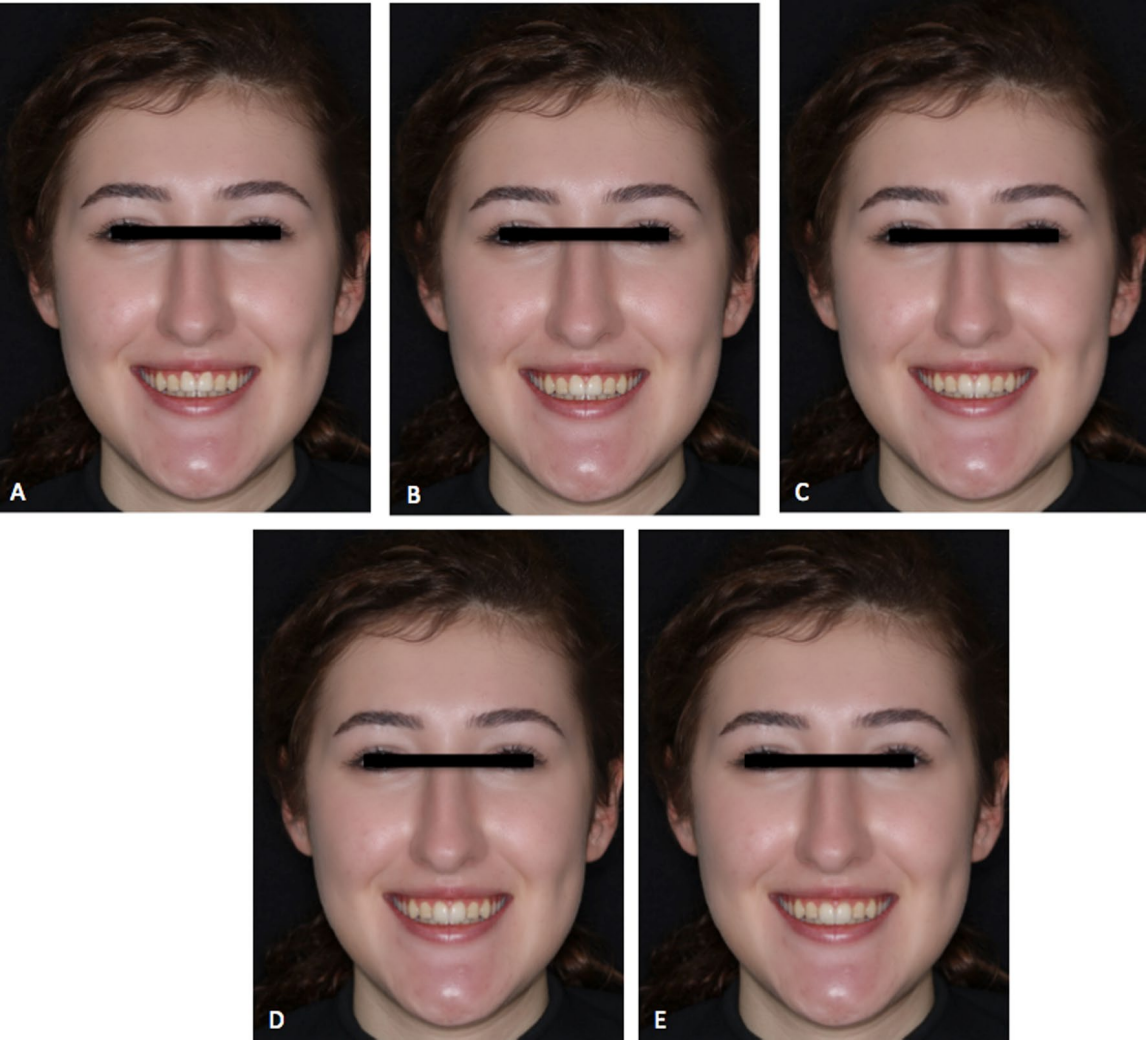
Full-face smile view in 0.5 mm altered vertical positions increments of a white woman showing increased vertical facial dimension. **A** − 1 mm; **B** − 0.5 mm; **C** 0 mm; **D** + 0.5 mm; **E** + 1 mm

In the present research, the face photographs to be evaluated by the participants in terms of esthetic perception were digitally processed using Adobe Photoshop (version CS5, Adobe Systems Inc, San Jose, Calif). By using the program, five separate photographs were obtained from the facial photographs of each patient. As a result, a total of 15 facial photographs were evaluated by each participant in terms of esthetic perception. The stains were removed, and color, brightness, and contrast were adjusted on the images. The right maxillary central incisor was used as a reference to achieve a magnification of 1:1 to correspond each millimeter of the image on A3 paper to each millimeter of the digital image of the patient. First of all, a standard smile was applied. On one side of the image, the gingival margins of the canine and central incisor were on the same line, and the gingival margin of the lateral incisor was 0.5 mm below this line. The incisal step between the central and lateral incisor was set to 1 mm. After these adjustments, and to make the image symmetrical, the adjusted side was duplicated. As shown in Table 1, the vertical position of the maxillary central incisor was changed (intruded or extruded) with 0.5 mm increments according to the reference gingival line between the central incisor and canine, which resulted in five images for each woman in a full-face view, yielding a total of 15 images. The upper limit of the full-face photograph was above the top of the head, and the lower limit was the base of the neck (Figs. 1, 2, and 3). The final images were presented in a standardized color and format with a resolution of 300 dots per inch (dpi). In the first stage, before evaluation, the standardized photographs of the patients with three different facial types were displayed simultaneously, and the raters were calibrated. After that, the five pictures for each facial type were shown randomly for the normal facial vertical dimension, the decreased facial vertical dimension, and the increased facial vertical dimension. The raters were not allowed to reevaluate the pictures.

**Table 1:** The vertical position definitions for smile evaluation used in the study

Altered vertical position for central incisors	Central incisor edge according to lateral incisor edge	Central incisor gingival margin according to the canine gingival margin	Gingival display amount
− 1 mm	Matching	1 mm above	0
− 0.5 mm	0.5 mm below	0.5 mm above	0.5 mm
0 mm (standard position)	1 mm below	Matching	1 mm
+ 0.5 mm	1.5 mm below	0.5 mm below	1.5 mm
+ 1 mm	2 mm below	1 mm below	2 mm

A computer-based questionnaire, constructed using Survey Monkey (Monte Carlo, CA), which linked questions to images, was used in the survey. It was comprised of two parts. This link was sent to the orthodontists and prosthodontists by e-mail and were given to the laypersons directly at the clinic. The first part included a descriptive survey about personal demographic characteristics, and the second included image-based questions on smile esthetic measurement. A visual analog scale (VAS) (100 mm long, with 0 being unattractive and 100 being very attractive) was placed below each smile to allow raters to evaluate the attractiveness of each smile independently. Each rater marked the point on the VAS to the best of their judgment. After that, the points marked on the VAS were converted into an esthetic score from 0 to 100 mm. In order to assess the intra-rater reliability of the measurements, 15 raters from each group (at least 25% of the raters) were randomly selected. They were asked to score the same 15 images after a four-week interval. The intra-class correlation coefficients calculated for each image ranged between 0.862 and 1.000 for orthodontists, between 0.926 and 1.000 for prosthodontists, and between 0.818 and 0.999 for laypersons. Consequently, the judgments of all three groups were found to be reliable.

### Statistical analysis

Statistical analysis was done using the IBM SPSS Statistics V23 program. Qualitative variables were summarized by frequency and percentage, whereas quantitative variables were summarized by mean, standard deviation, median, 25th percentile, and 75th percentile. Chi-square analysis was used to determine whether there was a significant difference between the two qualitative variables. The Pearson Chi-square test was used for crosstabs with an expected frequency of less than five cells not exceeding 20%, and the exact test for crosstabs exceeded. The distribution of quantitative variables was analyzed by the Kolmogorov–Smirnov test of normality and graphical methods (histogram, box-plot). One-way analysis of variance (one-way ANOVA) was used to determine whether there was a difference between more than two independent groups in terms of the quantitative variables. As a post hoc analysis, if the assumption of homogeneity of variance was met, the Bonferroni test was used; otherwise, the Tamhane test was used. Comparisons of more than two dependent groups were examined with repeated measures one-way ANOVA. The Bonferroni test was used as post hoc analysis for the variables found to be significant as a result of the analysis. The significance level was taken as 0.05 for all analyses.

## Results

The results of the demographic variable comparisons are presented in Table 2. The sample of evaluators was composed of 60 orthodontists (42 female and 18 male), 60 prosthodontists (36 female and 24 male), and 60 laypersons (30 female and 30 male). Gender distribution did not differ among the different rater groups (*p* = 0.082). The percentage of individuals between 24 and 30 years old was significantly less for orthodontists than for laypersons (*p* = 0.041). While the majority of orthodontists were working in the private sector (*N* = 33, 55%), the majority of prosthodontists (*N* = 31, 51.7%) were working in university hospitals. There was no significant difference between orthodontists and prosthodontists in terms of academic degree distribution (*p* = 0.743) and clinical experience years (*p* = 0.426).

**Table 2:** Demographic variable comparisons between different raters

Variables	Orthodontists	Prosthodontists	Laypersons	Difference *p* value
Number	*N* = 60	*N* = 60	*N* = 60	
Age range (years)				
24–30	12^a^ (20%)	23^a,b^ (38.3%)	28^b^ (%46.7)	0.041*
31–40	30^a^ (50%)	18^a^ (30%)	24^a^ (40%)	*p* > 0.05
41–50	9^a^ (15%)	10^a^ (16.7%)	5^a^ (8.3%)	*p* > 0.05
51–60	5^a^ (8.3%)	7^a^ (11.7%)	2^a^ (3.3%)	*p* > 0.05
> 60	4^a^ (6.7%)	2^a^ (3.3%)	1^a^ (1.7%)	*p* > 0.05
Gender (F/M)				
F	42 (70%)	36 (60%)	30 (50%)	0.082
M	18 (30%)	24 (40%)	30 (50%)	
Education status				
Undergraduate	0^a^ (0%)	0^a^ (0%)	27^b^ (45%)	< 0.001*
Degree	0^a^ (0%)	0^a^ (0%)	33^b^ (55%)	
Expertise	60^a^ (100%)	60^a^ (100%)	0^b^ (0%)	
Institution of employment				
University hospital	26^a^ (43.3%)	31^a^ (51.7%)	–	< 0.001*
Public hospital	0^a^ (0%)	11^b^ (18.3%)	–	
Private sector	33^a^ (55%)	18^b^ (30%)	–	
Retired	1^a^ (1.7%)	0^b^ (0%)	–	
Academic title				
Professor	6^a^ (10%)	4^a^ (6.7%)	–	0.743
Associate Professor	5^a^ (8.3%)	4^a^ (6.7%)	–	
Assistant Professor	11^a^ (18.3%)	8^a^ (13.3%)	–	
Dr.	38^a^ (63.3%)	44^a^ (73.3%)	–	
Years of clinical experience range (years)				
1–5	17^a^ (28.3%)	24^a^ (40%)	–	0.426
6–10	12^a^ (20%)	11^a^ (18.3%)	–	
11–20	18^a^ (30%)	10^a^ (16.7%)	–	
21–30	8^a^ (13.3%)	8^a^ (13.3%)	–	
> 30	5^a^ (8.3%)	7^a^ (11.7%)	–	

Pearson Chi-square test was used for comparison of the groups

Difference is significant at 0.05 level

*Means significant difference

Different letters (a and b) mean statistically significant differences

Based on the analysis of smiles within the full-face images for increased facial vertical height, the highest scores for orthodontists were given to the 0.5 mm extruded (64.18 ± 26.36) smiles, prosthodontists gave them to the control (57.28 ± 19.80) smiles, and the laypersons gave them to the 1 mm extruded (61.27 ± 25.98) smiles. The lowest scores were given to the smiles with a 1 mm intrusion for all rater groups, which were, respectively, 42.30 ± 21.59 for orthodontists, 50.12 ± 26.23 for prosthodontists, and 56.57 ± 26.80 for laypersons. Orthodontists gave statistically significant lower scores than laypersons for the smile with a 1 mm intrusion (*p* = 0.006) (Table 3).

**Table 3:** Scores for the attractiveness of the full-face images in increased facial vertical height and comparison of the scores between different raters

Smile variable	Orthodontists (1)		Prosthodontists (2)		Laypersons (3)		Difference *p* value
	Mean ± SD	Median (%25–%75)	Mean ± SD	Median (%25–%75)	Mean ± SD	Median (%25–%75)	
− 1 mm	42.30 ± 21.59	41.50 (27.50–52.50)	50.12 ± 26.23	45.50 (29.50–70)	56.57 ± 26.80	55 (40–75)	0.008* (1–3; *p* = 0.006)
− 0.5 mm	48.83 ± 19.16	47.50 (35.50–60.50)	55.40 ± 19.56	54.50 (43–67.50)	56.70 ± 24.33	55 (40–72.5)	0.095
0 mm	60.18 ± 17.46	60 (50–73)	57.28 ± 19.80	57.50 (40.50–70)	59.10 ± 23.39	52.50 (45–77.50)	0.733
+ 0.5 mm	64.18 ± 26.36	68.50 (50–79.50)	54.93 ± 25.12	52 (35–74.50)	59.82 ± 22.71	57.50 (40–77.50)	0.088
+ 1 mm	55.03 ± 22.23	51 (40.50–72)	54.88 ± 26.66	50 (32.50–80)	61.27 ± 25.98	60 (45–80)	0.088

One-way Anova was used for comparison of the groups. Pairwise comparisons for the variables found to be significant were made using the Bonferroni post-hoc test

Difference is significant at 0.05 level

*Means significant difference

1 = Orthodontists, 2 = prosthodontists, 3 = laypersons

1–2 = Comparison of 1 and 2; 2–3 = comparison of 2 and 3; 1–3 = comparison of 1 and 3

Table 4 shows the smile attractiveness scores for the full-face image with decreased facial vertical height. For all rater groups, the highest scores were obtained at the 0.5 mm intrusion, with an increasing pattern from orthodontist to laypersons (63.95 ± 22.08 for orthodontists, 79.87 ± 21.43 for prosthodontists, and 79.88 ± 19.17 for laypersons). Orthodontists gave significantly lower scores than prosthodontists (*p* = 0.032) and laypersons (*p* = 0.001) at the 0.5 mm intrusion smile level. The lowest scores were obtained at the 0.5 mm extrusion level for all rater groups, with an increasing pattern from orthodontists to laypersons (48.68 ± 19.46 for orthodontists, 57.48 ± 21.33 for prosthodontists, and 65.40 ± 21.48 for laypersons). The scores were significantly higher for laypersons at the 0.5 mm extrusion level when compared to orthodontists (*p* < 0.001) and prosthodontists (*p* = 0.001). When the smile attractiveness for the 1 mm intrusion and the 0 mm and 1 mm extrusion was compared between the groups, orthodontists gave statistically significant lower scores than laypersons gave (*p* < 0.001) for the full-face images with decreased facial vertical height.

**Table 4:** Scores for the attractiveness of the full-face images in decreased facial vertical height and comparison of the scores between different raters

Variable	Orthodontists (1)		Prosthodontists (2)		Laypersons (3)		Difference *p* value
	Mean ± SD	Median (%25–%75)	Mean ± SD	Median (%25–%75)	Mean ± SD	Median (%25–%75)	
− 1 mm	57 ± 18.99	60 (42.50–70.50)	60.43 ± 23.88	61.50 (44–75)	72.57 ± 22.47	75 (65–90)	< 0.001^a^* (1–3; *p* < 0.001) (2–3; *p* = 0.008)
− 0.5 mm	63.95 ± 22.08	65.50 (50–80)	79.87 ± 21.43	72.50 (61–86.50)	79.88 ± 19.17	85 (72.50–90)	0.001^a*^ (1–2; *p* = 0.032) (1–3; *p* = 0.001)
0 mm	63.83 ± 19.43	65.50 (50–79.50)	72.63 ± 79.64	75 (62–88.50)	76.52 ± 16.78	79 (70–89)	< 0.001^a*^ (1–3; *p* < 0.001)
+ 0.5 mm	48.68 ± 19.46	50 (37–59)	57.48 ± 21.33	56 (42.50–75)	65.40 ± 21.48	70 (52.50–80)	< 0.001^b*^ (3–1; *p* < 0.001) (3–2; *p* = 0.001)
+ 1 mm	58.72 ± 20.39	57 (46.50–74)	58.45 ± 27.44	60.50 (32.50–81)	75.05 ± 21.20	80 (67.50–90)	< 0.001^a*^ (1–3; *p* < 0.001)

One-way Anova was used for comparison of the groups. Pairwise comparisons for the variables found to be significant were made using the Bonferroni post hoc test

^a^Bonnferroni was used for post hoc test

^b^Tamhane was used for post hoc test

Difference is significant at 0.05 level

*Means significant difference

1 = Orthodontists, 2 = prosthodontists, 3 = laypersons

1–2 = Comparison of 1 and 2; 2–3 = comparison of 2 and 3; 1–3 = comparison of 1 and 3

Table 5 shows the smile attractiveness scores for the full-face image with normal facial height. All the three rater groups gave the highest scores for the 0 mm (control) smile design, which were 68.87 ± 18, 72.40 ± 21.24 and 83.33 ± 15.63 for orthodontists, prosthodontists, and laypersons, respectively. When these scores were compared among the groups, laypersons gave significantly higher scores compared to orthodontists (*p* < 0.001) and prosthodontists (*p* = 0.005). The lowest scores were given to the smiles with a 1 mm extrusion for orthodontists (53.70 ± 18.52) and prosthodontists (50.88 ± 18.40), while the laypersons gave the lowest scores to the smiles with a 0.5 mm extrusion (63.53 ± 25.84). At the level of 1 mm extrusion, scores given by the orthodontists were significantly lower than those given by the laypersons (*p* < 0.001). At the level of 1 mm intrusion, orthodontists gave lower scores than the prosthodontists (*p* = 0.007) and the laypersons (*p* < 0.001). At the level of the 0.5 mm intrusion, the highest scores were given by the laypersons, compared to the orthodontists (*p* < 0.001) and prosthodontists (*p* = 0.006).

**Table 5:** Scores for the attractiveness of the full-face images in normal facial height and comparison of the scores between different raters

Variable	Orthodontists (1)		Prosthodontists (2)		Laypersons (3)		Difference *p* value
	Mean ± SD	Median (%25–%75)	Mean ± SD	Median (%25–%75)	Mean ± SD	Median (%25–%75)	
− 1 mm	51.70 ± 19.21	50 (37.50–69.50)	62.95 ± 20.39	62 (49–79)	68.47 ± 20.62	72.50 (50–85)	< 0.001*^a^ (1–2; *p* = 0.007) (1–3;p < 0.001)
− 0.5 mm	63.13 ± 17.64	61.50 (50–76.50)	65.57 ± 17.74	70 (55–78.50)	76.15 ± 19.69	80 (67.50–90)	0.000*^a^ (3–1; *p* < 0.001) (3–2; *p* = 0.006)
0 mm	68.87 ± 18	70.50 (56.50–80)	72.40 ± 21.24	75 (56.50–90)	83.33 ± 15.63	87 (75–95)	0.000*^b^ (3–1; *p* < 0.001) (3–2; *p* = 0.005)
+ 0.5 mm	65.28 ± 18.80	69.50 (50–81.50)	61.88 ± 20.82	65 (42.77)	63.53 ± 25.84	70 (40–85)	0.645^b^
+ 1 mm	53.70 ± 18.52	50 (40–70)	50.88 ± 18.40	50 (39.50–62)	77.77 ± 20.74	80 (72.50–92.50)	< 0.001*^a^ (1–3; *p* < 0.001)

One-way Anova was used for comparison of the groups. Pairwise comparisons for the variables found to be significant were made using the Bonferroni post hoc test

^a^Bonnferroni was used for post hoc test

^b^Tamhane was used for post hoc test

Difference is significant at 0.05 level

^*^ Means significant difference

1 = Orthodontists, 2 = prosthodontists, 3 = laypersons

1–2 = Comparison of 1 and 2; 2–3 = comparison of 2 and 3; 1–3 = comparison of 1 and 3

The comparison of the different vertical dimensions with respect to the smile attractiveness of the full-face images for the orthodontists, prosthodontists, and laypersons is shown in Table 6.

**Table 6:** Comparison of different vertical dimensions with respect to smile attractiveness of the full-face images for the orthodontists, prosthodontists and laypersons

Smile variable	R	Normal facial height (1)		Increased facial vertical height (2)		Decreased facial vertical height (3)		Difference *p* value
		Mean ± SD	Median (%25–%75)	Mean ± SD	Median (%25-%75)	Mean ± SD	Median (%25–%75)	
− 1 mm	O	51.70 ± 19.21	50 (37.50–69.50)	42.30 ± 21.59	41.50 (27.50–52.50)	57 ± 18.99	60 (42.50–70.50)	< 0.001* (2–1; *p* = 0.022) (2–3; < 0.001)
	P	62.95 ± 20.39	62 (49–79)	50.12 ± 26.23	45.50 (29.50–70)	60.43 ± 23.88	61.50 (44–75)	0.006* (2–1; *p* = 0.009) (2–3; *p* = 0.049)
	L	68.47 ± 20.62	72.50 (50–85)	56.57 ± 26.80	55 (40–75)	72.57 ± 22.47	75 (65–90)	< 0.001* (2–1; *p* = 0.011) (2–3; *p* < 0.001)
− 0.5 mm	O	63.13 ± 17.64	61.50 (50–76.50)	48.83 ± 19.16	47.50 (35.50–60.50)	63.95 ± 22.08	65.50 (50–80)	< 0.001* (2–1;p < 0.001) (2–3; *p* < 0.001)
	P	65.57 ± 17.74	70 (55–78.50)	55.40 ± 19.56	54.50 (43–67.50)	79.87 ± 21.43	72.50 (61–86.50)	< 0.001* (2–1; *p* = 0.001) (2–3; *p* < 0.001)
	L	76.15 ± 19.69	80 (67.50–90)	56.70 ± 24.33	55 (40–72.5)	79.88 ± 19.17	85 (72.50–90)	< 0.001* (2–1; *p* < 0.001) (2–3; *p* < 0.001)
0 mm	O	68.87 ± 18	70.50 (56.50–80)	60.18 ± 17.46	60 (50–73)	63.83 ± 19.43	65.50 (50–79.50)	0.039* (1–2; *p* = 0.014)
	P	72.40v21.24	75 (56.50–90)	57.28 ± 19.80	57.50 (40.50–70)	72.63 ± 79.64	75 (62–88.50)	< 0.001* (2–1; *p* < 0.001) (2–3; *p* = 0.001)
	L	83.33 ± 15.63	87 (75–95)	59.10 ± 23.39	52.50 (45–77.50)	76.52 ± 16.78	79 (70–89)	< 0.001* (2–1; *p* < 0.001) (2–3; *p* < 0.001)
+ 0.5 mm	O	65.28 ± 18.80	69.50 (50–81.50)	64.18 ± 20.36	68.50 (50–79.50)	48.68 ± 19.46	50 (37–59)	0.119
	P	61.88 ± 20.82	65 (42–77)	54.93 ± 25.12	52 (35–74.50)	57.48 ± 21.33	56 (42.50–75)	0.315
	L	63.53 ± 25.84	70 (40–85)	59.82 ± 22.71	57.50 (40–77.50)	65.40 ± 21.48	70 (52.50–80)	0.001* (3–1; *p* = 0.030) (3–2; *p* < 0.001)
+ 1 mm	O	53.70 ± 18.52	50 (40–70)	55.03 ± 22.23	51 (40.50–72)	58.72 ± 20.39	57 (46.50–74)	0.127
	P	50.88 ± 18.40	50 (39.50–62)	54.88 ± 26.66	50 (32.50–80)	58.45 ± 27.44	60.50 (32.50–81)	0.200
	L	77.77 ± 20.74	80 (72.50–92.50)	61.27 ± 25.98	60 (45–80)	75.05 ± 21.20	80 (67.50–90)	< 0.001* (1–2; *p* = 0.001) (1–3; *p* = 0.003)

Multiple comparison with Bonferroni was used

Difference is significant at 0.05 level

*Means significant difference

R = Rater, O = orthodontists, P = prosthodontists, L = laypersons

1 = Normal Facial Height; 2 = Increased facial vertical height; 3 = Decreased facial vertical height

1–2 = Comparison of 1 and 2; 2–3 = comparison of 2 and 3; 1–3 = comparison of 1 and 3

Considering both the 1 mm and 0.5 mm intrusion of the maxillary central incisors, the increased facial vertical height images resulted in significantly lower scores than the normal and decreased facial vertical height images for all rater groups (*p* < 0.05). For the control (0 mm) smile design (1 mm vertical step between the central and lateral incisors), the orthodontists gave significantly higher scores for normal facial height than increased vertical facial height (*p* = 0.014). At the same level of the central incisor, both the prosthodontists and laypersons gave significantly lower scores for increased vertical facial height than normal and decreased vertical facial height (*p* < 0.001). For orthodontists and prosthodontists, scores at the level of 0.5 mm and 1 mm extrusion of the central incisors did not differ among the three different vertical facial height groups (*p* > 0.05). Considering the + 0.5 mm extrusion, however, the laypersons rated decreased vertical facial height significantly higher than normal (*p* = 0.03) and increased vertical facial height (*p* < 0.001). But when the central incisors extruded + 1 mm, laypersons gave higher scores for normal facial height than for increased and decreased vertical facial height (*p* < 0.001).

## Discussion

It is clear for almost many dentists that esthetic planning must begin within the noblest and most obvious area of the smile, known as “maxillary central incisors”. Based on the importance of the upper central incisors, numerous researchers [1, 4, 8, 9, 25, 26] have investigated and reported that the vertical occlusal height difference between the maxillary central and lateral incisor has an important impact on smile esthetic perception. However, none of these studies included prosthodontists as a rater group, and none evaluated the impact of vertical facial height on the smile esthetic. As it is known, the proper vertical positioning of the incisors in the anterior region is important for ideal esthetic restoration, veneer placement, and denture setting. The opinions of prosthodontists are also important, as are those of orthodontists [1]. Therefore, the purpose of our study was to determine the esthetic perceptions of orthodontists, prosthodontists, and laypersons regarding the different vertical position of central incisors in different facial vertical heights. One study [27] has reported that there are no significant differences in scores of perceptions when using full-face images or close-up images of smiles. For the present study, we chose to use full-face photographs of three female smiles taken with a natural head position to reveal the relationship between vertical facial dimensions and smile perception related to vertical position of the maxillary central incisors. One of the strongest aspects of our study was that all rater groups were completely similar in terms of gender distribution and almost similar in terms of age distribution. In addition, the fact that there was no difference between the dentistry rater groups (orthodontists and prosthodontists) in terms of academic degrees and years of clinical experience revealed the homogeneity of the groups in terms of demographic data.

When the vertical position of the maxillary central incisors is altered, both the design of the gingival margins and incisal edges are altered and must be evaluated together. Thus, in the present study, we followed the method suggested in the literature [1, 5] while modifying the maxillary central incisors’ vertical position while maintaining teeth anatomy and changing the gingival margins and incisal edges together.

According to this study’s results, for increased facial vertical height, the highest scores were assigned to + 0.5 mm extruded for the orthodontists, + 1 mm extruded for the laypersons, and 0 mm for the prosthodontists. However, at these three levels (0 mm, + 0.5 mm, and + 1 mm), the three rater groups did not differ from each other significantly. With the increase in facial height, orthodontists (1.5 mm) and laypersons (2 mm) found that the greater difference in vertical level between the central and lateral incisors was more aesthetic, while prosthodontists found the control smile (1 mm) more esthetic. The preference of greater steps between the central and lateral incisors is also confirmed by the studies of Machado et al. [1] and Ker et al. [25], with greater ideal step being 1.5 mm and 1.4 mm, respectively, in accordance with the findings for increased facial vertical height for the present study. The esthetic score results of our three rater groups for increased facial vertical height are also in agreement with other studies in the literature, which shows that a maxillary central-to-lateral incisor occlusal height difference of more than 1 mm is preferred [9, 28].

For the decreased facial vertical height smile, all rater groups attributed higher mean scores to the smile with − 0.5 mm intruded and the least mean scores to the smile with + 0.5 mm extruded. This result showed that a lower difference in vertical level between the central and lateral incisors (0.5 mm) was seen as more esthetic, whereas a higher difference in vertical level between the central and lateral incisors (1.5 mm) was seen as less esthetic when the facial vertical height decreased. For the higher scores, orthodontists gave significantly lower scores than prosthodontists and laypersons. This result may be attributed to the fact that orthodontists routinely use facial analysis and smile analysis during treatment planning and diagnosis [29–31], which makes them more critical for identifying smaller levels of deviations compared to other dentistry professionals and laypersons, as shown by previous studies [4, 12, 13, 15, 22]. Other studies have shown that orthodontists tend to be stricter in their evaluations and give lower mean scores [1, 4, 12, 13, 18].

While evaluating the smile attractiveness in normal facial height, the 0 mm smile (control) design was preferred as the most attractive smile feature for all rater groups. However, laypersons gave significantly higher scores than the prosthodontists and orthodontists. The main reason for this difference may be the influence of the level of dental knowledge and experience because dentists are trained regarding ideal smile features and would give smaller scores because they could detect even the slightest of variations.

Considering all facial heights, orthodontists and laypersons gave the highest scores to the vertical relationship of incisor borders as 1 mm step (control smile). Like the findings of the present study, Machado et al. [4] also found that the most accepted vertical relationship of incisor borders is the 1 mm step and that the scores of the orthodontists were lower than those of the laypersons. However, in contrast to our findings, the orthodontists and laypersons displayed no statistically significant difference for the most attractive smiles (the central incisor gingival margins matched the canine, and the central-to-lateral incisal step was 1.0–1.5 mm) in the study of Menezes et al. [5].

Clinically, orthodontists and prosthodontists need to determine the ideal and esthetic vertical position difference between the central and lateral incisor to ideally position the brackets on incisors and/or to make an ideal treatment planning before restoration of these teeth. Considering the present study’s results, the facial vertical height of the patient also should also be considered before treatment planning regarding the positions of central and lateral incisors. Especially for increased vertical facial height, the raters differed from each other while giving higher scores to the smiles. The results of the present study indicate that in increased vertical facial height, a 1.5 mm vertical height difference between the central and lateral incisor was found to be more attractive by orthodontists, whereas a 1-mm vertical difference was found to be more attractive by prosthodontists. Laypersons found a 2-mm vertical distance between central and lateral incisor to be more attractive for increased vertical facial height. In this regard, keeping the distance between the central and lateral incisors longer than 1 mm in individuals with increased facial vertical height may be important in terms of increasing patient satisfaction in terms of clinical esthetics. On the contrary, when we consider the results of the study, keeping the distance between the central and lateral incisors shorter than 1 mm may create a more esthetically acceptable result in individuals with short facial vertical height.

In previous studies [1, 12, 13, 22, 25, 27], the smiles used for esthetic evaluation were mainly from white female patients. To better compare the results of our study with the existing studies, only female patients were evaluated for their smiles. The present study has some limitations that need to be taken into account when interpreting its results. The study did not investigate the impact of other factors, such as age, gender, and ethnicity on the perception of smile esthetics. Therefore, one of the limitations of the present study could be the fact that obtaining the results in a defined population is highly questionable by causing difficulties to applicate the results to other populations because of ethnic and sociocultural variations. In addition, the impact of other dental factors, such as tooth color and shape, was not considered, which might have influenced the present findings. Moreover, due to the subjectivity of smile attractiveness perceptions and the results being based on averages, the use of this study’s findings in clinical setting may be problematic.

## Conclusions

Based on the findings of this observational computer-based questionnaire study, the following conclusions were drawn:

- The vertical facial height significantly affected the perception of smile esthetics.
- For increased facial vertical height, the highest scores for the orthodontists were given to the 0.5 mm extruded, for prosthodontists were given to the control, and for layperson were given to the 1 mm extruded central incisor position.
- For decreased facial vertical height, the highest scores were obtained at 0.5 mm intrusion, with an increasing pattern from orthodontists to laypersons.
- All of the three rater groups gave the highest scores for the 0 mm (control) smile design for normal facial vertical height. When these scores were compared among the groups, the laypersons gave significantly higher scores compared to the orthodontists and prosthodontists.

## Data Availability

The data supporting the findings of this research can be obtained directly from the authors of the study.
